# Musical Training Improves Audiovisual Integration Capacity under Conditions of High Perceptual Load

**DOI:** 10.3390/vision4010009

**Published:** 2020-01-24

**Authors:** Jonathan M. P. Wilbiks, Courtney O’Brien

**Affiliations:** Department of Psychology, University of New Brunswick, 100 Tucker Park Road, P.O. Box 5050, Saint John, NB E2L 4L5, Canada; courtney.obrien@unb.ca

**Keywords:** audiovisual integration, musical training, visual perception

## Abstract

In considering capacity measures of audiovisual integration, it has become apparent that there is a wide degree of variation both within (based on unimodal and multimodal stimulus characteristics) and between participants. Recent work has discussed performance on a number of cognitive tasks that can form a regression model accounting for nearly a quarter of the variation in audiovisual integration capacity. The current study involves an investigation of whether different elements of musicality in participants can contribute to additional variation in capacity. Participants were presented with a series of rapidly changing visual displays and asked to note which elements of that display changed in synchrony with a tone. Results were fitted to a previously used model to establish capacity estimates, and these estimates were included in correlational analyses with musical training, musical perceptual abilities, and active engagement in music. We found that audiovisual integration capacity was positively correlated with amount of musical training, and that this correlation was statistically significant under the most difficult perceptual conditions. Results are discussed in the context of the boosting of perceptual abilities due to musical training, even under conditions that have been previously found to be overly demanding for participants.

## 1. Introduction

The capacity limits of various perceptual and cognitive processes have been, and continue to be, important topics of study in psychological research. From the classic work of Miller [1] who established the “magic number” seven for the capacity of working memory, to Cowan’s work [2] on visual working memory capacity, quantifying these capacities is paramount in establishing the capabilities and limitations of the human brain. While Miller’s and Cowan’s work was unimodal in nature, more recent research has focused on how presentation of stimuli in multiple modalities can influence perception of individual stimuli. This includes numerous combinations of modalities, including the integration of auditory and visual information. Some of the earliest work on audiovisual integration was that of Sumby and Pollack [3], who found that speech perception in a noisy environment is made easier by being able to watch the speaker’s lips. The demonstration of stimuli in one modality promoting better perception in a different modality led to a burgeoning field of research, much of which will be discussed within this special issue of *Vision*.

Multisensory integration plays an important role in the way we perceive and interact with the world. The capacity to attend to a single stimulus in our noisy environment can be challenging but can be facilitated by the presentation of a simultaneous (or near-simultaneous) tone [4,5,6]. In a study by Van der Burg et al. [6] of the “pip and pop” effect, visual targets were presented among an array of similar distractor stimuli, in cases where it is normally very difficult to locate the target. They found that presenting a spatially uninformative auditory event drastically decreased the time needed for participants to complete this type of serial search task. These results demonstrated the possible involvement of stimulus-driven multisensory integration on attentional selection using a difficult visual search task. They also showed that presentation of a visual warning signal provided no facilitation in the pip-and-pop task, which indicates that the integration of an auditory tone with a specific visual presentation leads it to “pop out” of the display, allowing that particular visual presentation to be prioritized for further processing. Furthering the contribution of these results, additional research showed that the observed facilitatory effect was not due to general alerting, nor was it due to top-down cueing of the visual change [7]. Instead, they proposed that the binding of synchronized audiovisual signals occurs rapidly, automatically, and effortlessly, with the auditory signal attaching to the visual signal relatively early in the perceptual process. As a result, the visual target becomes more salient within its dynamic, cluttered environment.

While much of the early work in audiovisual integration involved considering how temporal (e.g., [8,9,10,11]) and stimulus congruency (e.g., [12,13,14]) factors play a role in one-to-one integration, recent attention has turned to quantifying the capacity of audiovisual integration. Van der Burg et al. [15] suggested that the capacity is strictly limited to a single item, while work from our lab has proposed that the capacity is flexible, can exceed one, and modulates based on numerous factors [16,17]. In this series of experiments, participants were presented with eight visual stimuli (dots) arranged along an imaginary circle. Each of these dots was initially black or white, and then a number of the dots (between one and four, dependent on trial) changed polarity from black to white, or vice versa. A total of 10 dot presentations occurred wherein a different subset of dots changed polarity, and on one of the dot presentations, a tone was presented, with the understanding that participants would be able to note which dots changed in synchrony with the tone through a process of audiovisual integration. Participants were asked to respond to a probed location, as to whether the dot in that location did (or did not) change in synchrony with the tone. Results from our work showed that participants were able to integrate more than one visual stimulus with an auditory stimulus, and that capacity modulated as a function of factors such as cross-modal congruency or dots being connected by real or illusory polygons. The specific improvement provided by audiovisual integration in these studies was confirmed through comparison of audiovisual conditions to visual cueing conditions ([16], experiment 5 [18], experiments 1b and 2b). In these conditions, rather than a tone, participants were presented with a spatially uninformative visual stimulus to indicate the dots changing at the critical presentation. These visual cues were found to be inferior to audiovisual integration trials, confirming the unique contribution of audiovisual integration.

In considering the limitations of audiovisual integration, we have found that at particularly short visual durations of presentation, capacity was severely limited. This was the case in Van der Burg et al.’s work [15], where the rates of presentation were set at 150 or 200 ms. We replicated their findings at 200 ms and extended the findings with a 700 ms condition that led to capacity exceeding one item. In the majority of our experimental variations, we found that average capacity estimates in our populations did not exceed one item at 200 ms. We also confirmed that at this duration of presentation, there is no significant modulation in visual N1 amplitude when more than one dot was changing, suggesting that participants were unable to successfully perceive the incoming visual stimuli, and were therefore also unable to integrate more than one visual stimulus with an auditory stimulus ([16], experiment 4). That said, it is the case that certain individuals did demonstrate the ability to integrate more than one item at the fastest rate of presentation. Putting aside the question of whether capacity can reliably exceed one item or not, our recent focus has been on understanding how other unimodal and multimodal processes may be able to account for a wide range of capacity differences between individuals. An initial investigation into this question revealed that we can account for as much as a quarter of the variation present in audiovisual integration capacity estimates by considering an individual’s ability to track multiple objects, their tendency for global precedence on a Navon task, and their susceptibility to location conflict and alerting on the Attention Network Test [19].

In seeking to go beyond these lab-based measures, and in attempting to account for more than a quarter of the variation in estimates of audiovisual integration capacity, the current research sought to examine a type of auditory expertise that may further contribute to an individual’s capacity: musical training. Lee and Nopenney [20] provided insight on how musicians’ brains are fine-tuned for predictive action based on audiovisual timing. The researchers hypothesized that, compared with non-musicians, musically-trained individuals should display a narrower window of temporal integration for audiovisual stimuli. Participants were asked to judge the audiovisual synchrony of speech sentences and piano music at 13 levels of stimulus-onset asynchronies, and musicians were found to have a narrower temporal integration window for music, but not for speech. These findings suggested that piano training narrows the temporal integration window selectively for musical stimuli, but not for speech. Bishop and Goebl [21] demonstrated that the ability to perceive audiovisual asynchronies is strengthened through musicianship, but most prominently when the asynchronous presentation involved the musician’s instrument (pianists performed best when showed asynchronous presentations of piano playing). Similarly, Petrini et al. [22] demonstrated the relationship between memory of learned actions and the ability to recall upon those memories of actions to make associations between visual stimuli and auditory signals, even when one of the stimuli is incomplete. For instance, when hearing a particular snare or cymbal sound combined with the visual image of the drumstick hitting the drum, experienced drummers would perform better than novices in replicating the required movement.

Another study hypothesized that musicians, compared with non-musicians, would have a significantly enhanced temporal binding window, with greater accuracy on audiovisual integration tasks [23]. When discussing facets of multisensory perception such as the temporal binding window, it is commonly held that a ‘wider’ window (e.g., binding stimuli that are more disparate in time) is indicative of inferior function. Participants were subjected to a sound-induced flash illusion paradigm (cf. [24]). Across the two groups, the musicians showed lower susceptibility to perceiving the illusory flashes, particularly at faster stimulus onset asynchronies (SOA) where the illusion is most likely to occur. The results also showed that non-musicians’ temporal binding window was 2–3 times longer than that of musicians. A study by Landry and Champoux [25] further illustrated that trained musicians are fine-tuned to have stronger perceptual abilities than non-musicians. They pointed out that musicians have more developed cortical areas in the regions that process tactile, auditory and visual information, due to the impact of years of experience and training on neuroplasticity.

Talamini et al. [26] sought to determine whether the working memory capacity of musicians is advanced compared to that of non-musicians. Digit span recollection relies on the articulatory rehearsal loop, the phonological buffer, and subvocalization. Concurrent articulation (rehearsing anything else verbally while attempting to memorize a series of numerical or alphabetical structures) has been shown to reduce working memory capacity. The digit span test was administered to test the working memory of musicians vs. non-musicians, with musicians performing better than non-musicians when no concurrent articulation was required (but with musicians performing worse when concurrent articulation was required). Further to the study of audiovisual processing in musicians, Proverbio et al. [27] found that musicians are not subject to the McGurk effect [28]—a perceptual illusion that incorrectly combines sound to visual stimuli—in the same way non-musicians are. This ability to selectively integrate (or in the case of the McGurk effect, not integrate) auditory and visual information, speaks further to the superiority of musicians in audiovisual processing.

Based on the extant literature, including some from our laboratory, it is clear that the ability to identify the location of dots that changed polarity at a specific moment is increased by the presentation of a simultaneous tone. The ability of individuals to integrate this tone with the visual stimuli presented is an example of multisensory modulation of vision. It is also clear that there are underlying abilities that may further modulate this capacity, and that one of these abilities that has been found to influence other forms of multisensory integration is having had musical training in one’s life. The current study seeks to examine whether an individual’s experience (or lack thereof) with musical training contributes to a greater capacity for integration of audiovisual information. We expect to find that audiovisual integration capacity will be increased for those individuals with a high level of musical experience, as compared to more musically naïve individuals. This hypothesis is based on the literature discussed above, which suggests that musical training leads to an improvement in performance on both auditory and audiovisual tasks.

## 2. Method

### 2.1. Participants

The participants of this study were drawn from undergraduate psychology courses at the University of New Brunswick Saint John after being recruited through an undergraduate research participation pool. All recruitment and experimental practices were approved by the Research Ethics Board at the University of New Brunswick Saint John (Protocol #029-2018). Through conducting *a priori* power analyses based on findings in previous experiments, it was found that analyzing data from 20 participants would allow for a power (1—β) of 0.80, assuming α = 0.05. The decision was taken to initially test 20 participants before conducting a data management procedure as presented below. Additional participants were then tested until we had 20 participants whose data were usable for analysis. Informed, written consent was received from each of the participants prior to the experiment. Before data analysis, we calculated a 95% confidence interval around 50% (chance responding), as was done previously [16,17,18,19]. Five participants who fell within this confidence interval throughout all four blocks were then removed from the data set and replaced with data from new participants. The 20 participants who were considered for data analysis had a mean age of 19.2 years (SD = 1.8), of which 18 participants identified as female, two as male, and three people reported being left handed. While we acknowledge that removal of 20% of the individuals tested is not ideal, we also wished to maintain consistency with our data management practices used in earlier research projects. The reason so many participants failed to complete the task correctly in this case is likely a combination of the difficulty of the task, along with the fact that our sample was drawn completely from undergraduate student populations.

### 2.2. Materials

Visual stimuli were presented on a Dell 2407WFP monitor at a screen resolution of 1440 × 900 and a refresh rate of 60 Hz, using an Optiplex 755 Dell PC running Windows XP Professional (Service Pack 3; Dell, Austin, Texas) at a viewing distance of approximately 57 cm. Auditory stimuli were presented binaurally via Sennheiser HD280 PRO headphones (Sennheiser, Wennebostel, Germany). Stimulus presentation was controlled by Presentation (NBS) version 20.1, build 12.04.17, and behavioural responding was recorded with a Dell L100 keyboard. Eight dots (1.5° in diameter) could be displayed in one of two colors: Black (0, 0, 0) or white (255, 255, 255) against a mid-grey background (128, 128, 128). The main phase of the experiment consisted of the simultaneous display of eight dots along an implied circle (13° in diameter), the center of which was marked by a 0.15° fixation dot. A single, smaller probe dot was overlaid on a target dot at the end of each trial and was red (255, 0, 0) with a diameter of 1°. The auditory stimulus used was a 60-ms long, 400-Hz tone, with 5-ms linear onset and offset ramps, presented at an intensity of approximately 74 dB(C), which was created using SoundEdit 16 (MacroMedia, San Francisco, California).

The musical abilities of participants were quantified by completion of the Goldsmith Musical Sophistication Index (Gold-MSI; [29]). This is a self-report questionnaire that is designed to assess the musicality of individuals through a series of questions about their previous musical experience, training, as well as current level of engagement with music. It produces an overall score, as well as five sub scores: Active engagement, perceptual abilities, musical training, emotions, and singing abilities. While the full survey was administered to the participants, for the purposes of this experiment we were only interested in scores on the active engagement, perceptual abilities, and musical training subscales. The Gold-MSI is a highly reliable measure, with Cronbach’s α for active engagement of 0.872, for perceptual abilities of 0.873, and for musical training of 0.903, and test–retest correlations of 0.899, 0.894, and 0.974, respectively.

### 2.3. Procedure

**Figure 1:**
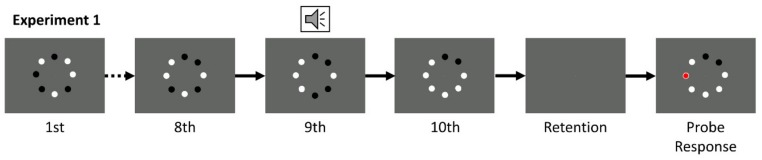
Trial schematic for the audiovisual integration capacity task. Participants saw a total of 10 visual presentations, with one accompanied by a tone. In this trial, the probed dot was one that did change at the presentation accompanied by a tone.

Twelve individual stimulus conditions were created by orthogonally varying the presentation duration of visual stimuli (200, 400, and 600 ms), and the number of visual stimuli that changed on each alternation (1, 2, 3, 4). These 12 conditions were each presented four times to create an experimental block. Each participant completed one randomized practice block of 16 trials, and eight experimental blocks, for a total of 384 experimental trials. Trial order was randomized in practice and in experimental trials, and Figure 1 provides a schematic of the experimental procedure. Each trial began with a fixation point displayed in the center of the screen for 500 ms. The sets of black and white dots were generated independently for each trial, and there was no restriction on which dot(s) could change color at each alternation, nor was there a restriction on how many dots could be white or black at any one time. An initial set of dots was presented, followed by nine additional dot presentations (for a total of ten presentations), with each set displayed for the amount of time specified as the presentation duration for that trial. The critical presentation was the penultimate (9th) frame. On this presentation, the onset of the dots was accompanied by an auditory tone. Following a final (10th) presentation, a 1000 ms retention interval occurred during which only the fixation point was displayed on the screen. During the recall phase, the tenth array of dots was displayed again, with the same locations black and white as when it was first presented, along with an overlay of a red probe dot on one of the eight dots. Participants were asked to respond to whether the dot at the probe location had changed or not on the critical display (i.e., the change from the 8th to 9th display) by pressing the number 1 on the number pad if that dot did not change, and by pressing the number 2 on the number pad if that dot did change. The probe had a validity of 50%, and the location for invalid trial probes was randomly determined. No feedback was provided, and the subsequent trial began shortly after a response was entered.

## 3. Results

We calculated the proportion correct for each combination of conditions and for each participant, and then subjected the raw data to a model-fitting procedure analogous to Cowan’s *K* [2]. The data for this experiment are available at: https://osf.io/g3ks2/. According to this model, if the number of visual stimuli changing (*n*) is less than or equal to an individual’s capacity (*K*), the probability of successful integration approaches certainty (i.e., if *n* ≤ *K*, then *p* = 1). Under conditions where an individual’s capacity is less than the number of visual stimuli changing (i.e., *n* > *K*), the probability of integration is modelled based on the equation: *p* = *K*/2*n* + 0.5. The fitting procedure involves using *K* as the free parameter and optimizing this value to minimize root-mean-square error between the raw proportion of correct responses for each number of locations changing and the ideal model. As the fitting procedure uses the values for each number of locations changing, along with the proportion of correct responses for each condition, capacity estimates (*K*) are obtained for each duration of presentation with no further consideration of number of locations changing or proportion correct responding.

**Figure 2:**
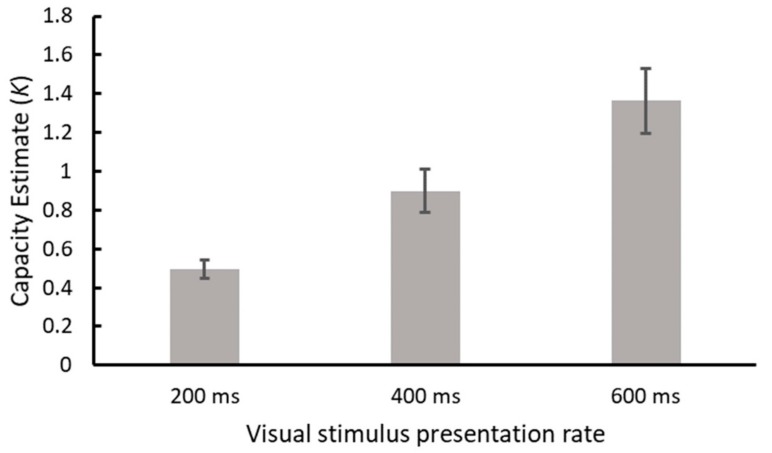
Capacity estimates (*K*) for each duration of visual stimulus presentation. Error bars represent standard error of the mean.

An initial analysis was conducted by means of one-way ANOVA examining capacity estimates for each duration of presentation. A significant main effect of presentation duration was observed, F(2,38) = 29.951, MSE = 0.126, *p* < 0.001, η_p_^2^ = 0.612, indicating an increase in capacity with slowing rates of presentation. Means and standard errors are displayed in Figure 2 and post hoc testing with Tukey’s HSD (*p* < 0.05) confirmed that each level of presentation duration yielded a capacity estimate that was significantly different from the others. Having established that capacity in this sample increased as a function of visual stimulus presentation duration, we subsequently determined whether an individual’s level of musical training was correlated with their capacity estimates. Capacity measures for each presentation duration (200, 400, and 600 ms) were entered into Spearman correlations with the following sub scores from the Goldsmith MSI: Active engagement, perceptual abilities, and musical training. Spearman correlations were used as the data were found to be non-parametric, and a Bonferroni-adjusted significance cut-off of *p* < 0.017 was employed, due to each score being used for three comparisons (0.05/3 ≈ 0.017) Full results of the correlations are displayed in Table 1. The data revealed that there was only one correlation that reached standard levels of statistical significance, which was between capacity at 200 ms and the musical training sub score (*r* = 0.536, *p* = 0.015). We expected to find significant correlations between capacity estimates and the other subscales of the MSI, but we will discuss further in the following section why this result is particularly interesting.

## 4. Discussion

The purpose of this study was to determine whether the modulation of visual perception through audiovisual integration would be further augmented by musical training. Our initial finding confirmed what we have found previously—that audiovisual integration capacity is flexible and increases as a function of slower rates of presentation. Our findings with regard to the interaction between capacity and musical training were more nuanced. Correlational analysis revealed that higher levels of musical training were associated with higher estimates of audiovisual integration capacity, specifically for the fastest (200 ms) duration of visual presentation. There was a small positive correlation between musical training and integration capacity at the other presentation rates, but only at 200 ms was the correlation a statistically significant one. While this was not exactly the pattern of results that we had expected, it is certainly one worth discussing. In our previous work, integration capacity at 200 ms was only able to exceed one item when it was further boosted by perceptual chunking of visual locations [17]. If individuals with higher levels of musical training have greater capacity estimates at 200 ms than individuals with less musical training, it would be worthwhile to recruit a group of highly trained musicians to see if this training allows them to reliably integrate more than one item at this relatively short duration of presentation. Previous research has shown that presenting stimuli with a greater perceptual load as indexed by number of stimuli [30,31] or presentation rate [32] leads to decreases in performance on tasks related to perception. Our previous work has shown that reducing the effective number of items to be tracked leads to an increase in capacity estimates [17,18], suggesting that perceptual load plays a similar role in audiovisual integration. Additionally, our previous research with electrophysiological recording showed that participants were unable to process incoming information sufficiently at a presentation duration of 200 ms, as indexed by a lack of modulation in N1 amplitude [16]. While perceptual load seems to serve as a limiting factor in an individual’s capacity to integrate auditory and visual information, the current data suggest that musical training may allow an individual to overcome this challenge. This could be confirmed in future studies by conducting an EEG study on trained musicians, compared to a group of non-musicians, and comparing components such as the N1 or contralateral delay activity (CDA [31]).

The “pip-and-pop” effect [6], on which the current research is loosely based, is described as being automatic, rapid, and effortless in individuals being presented with stimuli. That is to say, it occurs outside of the individual’s conscious understanding, at a perceptual level. This is in accord with what we have found previously in both behavioural [17,18] and electrophysiological [16] work. While this effect may well be automatic, it is also likely that previous experience and expertise would have an effect on an individual’s ability to integrate auditory and visual information. In recently completed work [19], we determined that a number of perceptual and cognitive functions can be shown to contribute to individual differences in audiovisual integration capacity. While these relatively low-level effects have been found to play a role, the current research has shown that higher level factors such as lifelong training can also play a role. While this is the first such experiment to show differences in audiovisual integration capacity, there is previous literature showing similar differences on perception of audiovisual synchrony perception [21], width of temporal binding window [23], McGurk susceptibility [27], and working memory capacity [26].

It would be of interest, moving forward, to employ more ecologically valid stimuli, as it has been shown that experiments using artificial tones do not generalize to natural sounds [33,34]. For example, the current experiment used a pure tone (single frequency) but it would be of additional interest to use intermediate types of tones, such as instrumental tones and other ecologically valid stimuli, which might also further promote the advantage of musicians, as they would likely have more experience with such stimuli through their practice as a musician. This would also be in alignment with work from Lee and Noppeney [20] who used musical stimuli in their experiments and found that musically trained individuals had a narrower (that is, superior) temporal binding window than those without musical training. Our findings showed limited differences between individuals with relatively greater or lesser amounts of musical training on an audiovisual integration capacity task using abstract stimuli (sine tones and dots). It follows that we may observe greater differences based on musical training if we were to employ more ecologically valid stimuli.

The current experiment is part of an ongoing program of research in our lab in which we are seeking to identify factors that may influence variability in audiovisual integration capacity. In addition to the previously completed research on underlying perceptual and cognitive factors [19], work is underway to examine differences stemming from variability in traits related to autism spectrum disorder, as well as major depressive disorder. This work is currently in the initial stages, but data will initially be used in building an increasingly efficient predictive model of audiovisual integration capacity. Having done so, it will be of interest to consider applications of audiovisual integration capacity as a potential early diagnostic system for autism, as the sensory and perceptual differences in autism can, in many cases, be observed earlier than traditional diagnostic systems can be employed. This is of utmost importance in the field of autism spectrum disorder, as earlier diagnosis of ASD has been associated with better treatment outcomes in the long term [35]. The results of the current experiment, however, add to the corpus of literature showing that vision can be affected positively by other sensory inputs, and that this phenomenon can be further modulated through the lifetime experience of an individual.

## Figures and Tables

**Table 1:** Results for the Spearman correlation between estimates of audiovisual integration capacity (*K*) and sub scores on the Goldsmith MSI. Significance is flagged at a Bonferroni-adjusted *p* < 0.017.

	Active Engagement		Perceptual Abilities		Musical Training	
Presentation Duration	*r*	*p*	*r*	*p*	*r*	*p*
200 ms	0.380	0.099	0.125	0.600	0.536	0.015
400 ms	0.449	0.047	0.007	0.976	0.295	0.207
600 ms	0.294	0.208	0.032	0.894	0.221	0.348

## Data Availability

The data for this experiment are available at: https://osf.io/g3ks2/.

## References

[1] Miller G.A. (1956). The magical number seven, plus or minus two some limits on our capacity for processing information. Psychol. Rev..

[2] Cowan N. (2001). The magical number 4 in short-term memory: A reconsideration of mental storage capacity. Behav. Brain Sci..

[3] Sumby W.H., Pollack I. (1954). Visual contribution to speech intelligibility in noise. J. Acoust. Soc. Am..

[4] Bertelson P., Pavani F., Ladavas E., Vroomen J., De Gelder B. (2000). Ventriloquism in patients with unilateral visual neglect. Neuropsychologia.

[5] Vroomen J., De Gelder B. (2000). Sound enhances visual perception: Cross-modal effects of auditory organization on vision. J. Exp. Psychol. Hum..

[6] Van der Burg E., Olivers C.N., Bronkhorst A.W., Theeuwes J. (2008). Pip and pop: Nonspatial auditory signals improve spatial visual search. J. Exp. Psychol. Hum..

[7] Van der Burg E., Cass J., Olivers C., Theeuwes J., Alais D. (2010). Efficient visual search from synchronized auditory signals requires transient audiovisual events. J. Vis..

[8] Zampini M., Shore D.I., Spence C. (2003). Audiovisual temporal order judgments. Exp. Brain Res..

[9] Spence C., Squire S. (2003). Multisensory integration: Maintaining the perception of synchrony. Curr. Biol..

[10] Van Wassenhove V., Grant K.W., Poeppel D. (2007). Temporal window of integration in audio-visual speech perception. Neuropsychologia.

[11] Soto-Faraco S., Alsius A. (2009). Deconstructing the McGurk-MacDonald illusion. J. Exp. Psychol. Hum..

[12] Evans K.K., Treisman A. (2010). Natural cross-modal mappings between visual and auditory features. J. Vis..

[13] Spence C. (2011). Crossmodal correspondences: A tutorial review. Atten. Percept. Psychophys..

[14] Leboe L.C., Mondor T.A. (2007). Item-specific congruency effects in nonverbal auditory Stroop. Psychol. Res..

[15] Van der Burg E., Awh E., Olivers C.N. (2013). The capacity of audiovisual integration is limited to one item. Psychol. Sci..

[16] Wilbiks J.M.P., Dyson B.J. (2016). The dynamics and neural correlates of audio-visual integration capacity as determined by temporal unpredictability, proactive interference, and SOA. PLoS ONE.

[17] Wilbiks J.M.P., Dyson B.J. (2018). The contribution of perceptual factors and training on varying audiovisual integration capacity. J. Exp. Psychol. Hum. Percept. Perform..

[18] Wilbiks J.M.P., Pavilanis A.D.S., Rioux D.M. (2020). Audiovisual integration capacity modulates as a function of illusory visual contours, visual display circumference, and sound type. Atten. Percept. Psychophys..

[19] Wilbiks J.M.P., Beatteay A. Predicting audiovisual integration capacity through multiple object tracking, orienting attention, and global precedence in perception. Proceedings of the Canadian Psychological Association.

[20] Lee H., Noppeney U. (2011). Long-term music training tunes how the brain temporally binds signals from multiple senses. Proc. Natl. Acad. Sci. USA.

[21] Bishop L., Goebl W. (2014). Context-specific effects of musical expertise on audiovisual integration. Front. Psychol..

[22] Petrini K., Russell M., Pollack F. (2008). When knowing can replace seeing in an audiovisual integration of actions. Cognition.

[23] Bidelman G. (2016). Musicians have enhanced multisensory binding: Experience-dependent effects in the double-flash illusion. Exp. Brain Res..

[24] Shams L., Kamitani Y., Shimojo S. (2002). Visual illusion induced by sound. Cogn. Brain Res..

[25] Landry S.P., Champoux F. (2016). Musicians react faster and are better multisensory integrators. Brain Cogn..

[26] Talamini F., Carretti B., Grassi M. (2016). The working memory of musicians and nonmusicians. Music. Percept..

[27] Proverbio A.M., Massetti G., Rizzi E., Zani A. (2016). Skilled musicians are not subject to the McGurk effect. Sci. Rep..

[28] McGurk H., MacDonald J. (1976). Hearing lips and seeing voices. Nature.

[29] Müllensiefen D., Gingras B., Musil J., Stewart L. (2014). Measuring the facets of musicality: The Goldsmiths Musical Sophistication Index (Gold-MSI). Personal. Individ. Differ..

[30] Lavie N. (2005). Load theory of selective attention and cognitive control. Trends Cogn. Sci..

[31] Drew H., Horowitz T.S., Vogel E.K. (2013). Swapping or dropping? Electrophysiological measures of difficulty during multiple object tracking. Cognition.

[32] Marois R., Ivanoff J. (2005). Capacity limits of information processing in the brain. Trends Cogn. Sci..

[33] Schutz M., Vaisberg J.M. (2014). Surveying the temporal structure of sounds used in Music Perception. Music. Percept..

[34] Schutz M., Gillard J. Generalizing audio-visual integration: What kinds of stimuli have we been using?. Proceedings of the International Multisensory Research Forum.

[35] Fernell E., Eriksson M.A., Gillberg C. (2013). Early diagnosis of autism and impact on prognosis: A narrative review. Clin. Epidemiol..

